# Foam Sclerotherapy for a Symptomatic Hepatic Cyst: A Preliminary Report

**DOI:** 10.1007/s00270-013-0761-5

**Published:** 2013-10-30

**Authors:** Chihiro Itou, Jun Koizumi, Takeshi Hashimoto, Kazunori Myojin, Tatehiro Kagawa, Tetsuya Mine, Yutaka Imai

**Affiliations:** 1 Department of Diagnostic Radiology, Tokai University School of Medicine, 143 Shimokasuya, Isehara, 259-1193 Japan; 2 Department of Diagnostic Radiology, Kouseikai Hosipital, Azuma-cho, Toyohashi, 440-0045 Japan; 3 Department of Diagnostic Radiology, Tokai University Oiso Hospital, 21-1 Gekkyo, Oiso-machi, Naka-gun, Kanagawa Japan; 4 Department of Gastroenterology and Hepatology, Tokai University School of Medicine, 143 Shimokasuya, Isehara, 259-1193 Japan

**Keywords:** Hepatic cyst, Sclerotherapy, Polidocanol foam, Carbon dioxide, C-arm CT

## Abstract

**Purpose:**

We evaluated our initial experience of performing sclerotherapy for symptomatic hepatic cysts using polidocanol foam instead of a liquid sclerosant.

**Methods:**

Three consecutively registered patients with symptomatic hepatic cysts (one with polycystic liver disease) underwent polidocanol foam sclerotherapy. A pigtail catheter was inserted into the targeted cyst following percutaneous cyst puncture under ultrasound guidance, and the cyst fluid was aspirated. To confirm the absence of communications between the cyst and surrounding hepatic vessels, 3 % polidocanol foam sclerosant was injected at a 1:4 ratio of polidocanol to air following digital subtraction cystography with carbon dioxide. C-arm computed tomography (CT) guidance also was used to monitor foam filling of the targeted cyst. The maximum dose of sclerosant injected per treatment session did not exceed 10 ml. The catheter was kept unclamped overnight for open drainage, and additional sclerotherapy sessions were performed on subsequent days, if needed.

**Results:**

Efficient sclerotherapy was achieved with an average of two sessions. The initial mean cyst volume was 1,052 ml, and gradual resolution was observed without recurrence. The mean reduction rate was 97.9 % (97.7–98.3 %), and all cyst-associated symptoms disappeared. The median follow-up period was 17 (range 6–21) months. Although one patient experienced moderate-grade fever and another moderate pain at the puncture site, no major complications were observed.

**Conclusion:**

Polidocanol foam sclerotherapy is a safe and effective treatment for symptomatic hepatic cysts.

## Introduction

Liquid sclerosants have been studied as alternatives to ethanol during sclerotherapy for symptomatic hepatic cysts [1–3], because systemic spillage or leakage of injected ethanol from a cyst can cause severe complications [4] and because ethanol sclerotherapy for polycystic liver disease (PLD) has a relatively high recurrence rate [5]. Five percent ethanolamine oleate iopamidol (EOI) is a feasible sclerosant for symptomatic hepatic cysts and has higher efficacy and lower complication rates than ethanol [6, 7]. However, liquid sclerosants may mix with the cyst contents and reduce the sclerotic effect. Foam sclerotherapy was introduced in the mid-1990s for treating lower extremity varicosities under ultrasound (US) guidance [8–10] and has since become more widely used for peripheral vascular malformations [11, 12] and for balloon-occluded retrograde transvenous obliteration of gastric varices [13].

The present study was designed to evaluate retrospectively the safety and efficacy of polidocanol foam sclerotherapy for symptomatic hepatic cysts.

## Materials and Methods

**Fig. 1:**
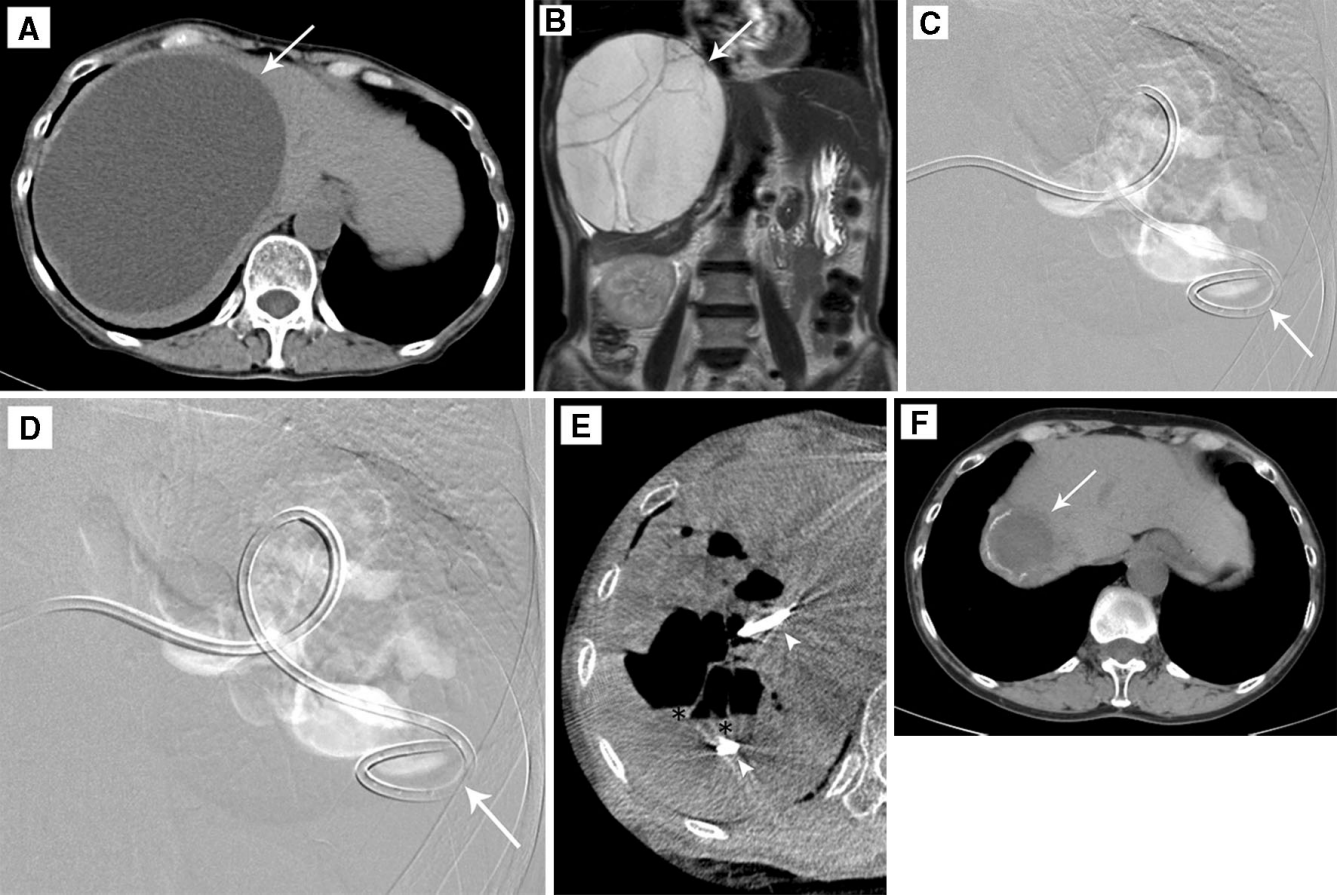
A 67-year-old woman (Case 1) with a symptomatic hepatic cyst causing abdominal distension. CT scan (*arrow*, **A**) shows a huge cyst in the right lobe of the liver, which is depicted as a well-demarcated cystic mass with several septums projecting to the right diaphragm on a coronal T2-weighted magnetic resonance image (*arrow*, **B**). While the patient was in a prone position, 10 ml of polidocanol foam was injected under fluoroscopy (**D**) through a pigtail catheter (*arrow*, **C**, **D**) placed in the cyst. This was performed after digital subtraction cystography (**C**) with 10 ml of CO_2_ in the same position. The distribution of the foam sclerosant was almost identical to that of CO_2,_ without any evidence of migration to the systemic circulation or surrounding hepatic vessels. **E** Axial reformation C-arm CT images depict the foam sclerosant as air density area distributed on the upper side within the cyst without any mixture or dilution and with a small amount of residual cyst fluid (*asterisk*) after percutaneous aspiration in the supine position. Note the pigtail catheter in the targeted cyst (*arrowheads*). **F** CT performed 6 months after sclerotherapy showing that the cyst was remarkably reduced in size (*arrow*)

This study was approved by the institutional review board of our hospital and was conducted after obtaining informed consent from all patients. Three consecutive patients with symptomatic hepatic cysts who were referred for interventional radiology between March 2010 and March 2012 were included. Hepatic cysts were detected and properties of the cyst walls and contents evaluated by US, contrast-enhanced computed tomography (CT) (Fig. 1A) or magnetic resonance (MR) (Fig. 1B). The presence and severity of any extrinsic compression of the surrounding hepatic vessels by the cysts also were determined. Initial cyst size and volume were determined by manual volumetry on CT.

Percutaneous cyst puncture through normal hepatic parenchyma was performed using a 19-gauge, 20-cm needle (Elaster Needle, Hakko, Japan) under US guidance after local anesthesia administration. Contact was confirmed by cystic fluid aspiration and by digital subtraction cystography with 10 ml of iodized contrast medium. An 8.5-F pigtail catheter (Ultrathane^®^ Bile Drainage Catheter; COOK, Bloomington, IN) was then introduced into the cyst through a 9-F peel-away introducer sheath (Peel-Away^®^ introducer Set; COOK) over a 0.038-in. guidewire, and the cyst contents were aspirated. The aspirated fluid volume was measured, and the specimens were examined bacteriologically and cytologically.

Absence of communications with the surrounding bile ducts or hepatic blood vessels and leakage into the intraperitoneal space was confirmed by digital subtraction cystography with 10 ml of carbon dioxide (CO_2_) (Fig. 1C) before polidocanol foam sclerotherapy.

Polidocanol foam was prepared by mixing 2 ml of 3 % polidocanol (Polidocasklerol, Zeria Pharmaceutical) with 8 ml of room air [8]. Foam was injected into the targeted cyst through the catheter, and fluoroscopy confirmed the absence of foam migration into surrounding hepatic vessels (Fig. 1D). The patient was moved from a lateral decubitus to another lateral decubitus or prone position after the each injection of 10 ml of polidocanol foam to ensure uniform contact between the cyst wall and sclerosant. C-arm CT (Axiom Artis FD system and DynaCT, Siemens Healthcare; Fig. 1E) was performed to confirm the accumulation of the foam–gas mixture in the targeted cyst and the absence of migration into the surrounding hepatic vessels or leakage into the intraperitoneal space. The catheter was left unclamped overnight for open drainage and to promote further cyst collapse, after the catheter was clamped for 30 min following foam injection.

The maximum dose of 3 % polidocanol per treatment session was 10 ml (equivalent to 50-ml foam). Sclerotherapy was repeated on subsequent days, if needed, on the basis of daily drainage from the catheter and the initial cyst volume. The catheter was removed 24 h after the final sclerotherapy session.

The total sclerosant dose used for each session and the presence and severity of any complications were documented. The targeted cyst volume was calculated by CT before sclerotherapy and routinely performed 1 or 6 month(s) after sclerotherapy.

## Results

The mean patient age was 71 years (range 67–75 years; Table 1). The investigated hepatic cysts included a simple solitary cyst, simple multiple cysts, and PLD-associated cysts. Clinical symptoms included abdominal distention, portal hypertension complicated by rectal varices, and PLD-induced obstructive jaundice with acute cholangitis. The mean initial cyst volume was 1,052 ml. In all cases, cytological examination of the aspirated cyst fluid confirmed the absence of malignancy, and bacteriological examinations did not detect any infection.

**Table 1:** Characteristics of patients with symptomatic hepatic cysts

Patient	Age (year)/sex	Symptoms	Etiology	Size (cm)	Estimated cyst volume (ml)	Aspirated volume (ml)	Amount of injected polidocanol/foam (ml)	Follow-up period (months)
1	67/F	Abdominal distention	Simple solitary cyst	15.4 × 11.7 × 15.3	1,585	1st.: 1380	1st.: 10/50	6
						2nd.: 20	2nd.: 10/50	
						3rd.: 20	3rd.: 10/50	
2	75/F	Portal hypertension	Simple multiple cysts,	8.1 × 6.7 × 6.9	181	1st: 150	1st.: 6/30	21
			Cirrhosis (hepatitis C)			2nd: 75	2nd.: 4/20	
3	71/M	Obstructive jaundice	Polycystic liver disease	17.1 × 11.4 × 14.5	1,390	895	8/40	17

The first two cases required two or three consecutive sessions of polidocanol foam sclerotherapy, but the last case only required a single session. The mean sclerosant dose used was 9.6 (range 4–10) ml per session, which corresponds to 3 % polidocanol. The total treatment dose of sclerosant required for sufficient sclerotherapy averaged 16 (range 8–30) ml per patients.

Table 2 presents sclerotherapy-related complications and changes in the targeted cyst volumes after sclerotherapy. All patients underwent follow-up CT within 6 months of sclerotherapy. Cyst volumes reduced gradually; the mean reduction rate 6 months after sclerotherapy was 97.9 % (97.7–98.3 %). Cyst-associated symptoms disappeared in all patients after sclerotherapy without any recurrence.

**Table 2:** Results and complications of polidocanol foam sclerotherapy for symptomatic hepatic cysts

Patient no.	Cyst volume (ml) (reduction rate)					Complications
	Initial	1 month follow-up	6 months follow-up	17 months follow-up	21 months follow-up	
1	1,585	282 (82.2 %)	35 (97.8 %)			Mild fever. No treatment required.
2	181		3 (98.3 %)		2 (98.9 %)	Fever of >38 °C. An antipyretic required.
3	1,390	36 (97.4 %)	32 (97.7 %)	25 (98.2 %)		Moderate pain at puncture site. An analgesic required.

One patient (Case 2) had a fever of >38 °C and required an antipyretic after the second sclerotherapy session, but fever subsided the following day. Another patient (Case 3) experienced moderate pain at the puncture site for several days, which was relieved by intravenous administration of an analgesic. No patient experienced no life-threatening complications.

## Discussion

Foam formulations of polidocanol mixed with room air or CO_2_ are used commonly to treat varicose veins in the extremities because several randomized trials have shown higher efficacy rates and lower recurrence rates with foam than with liquids [9, 10]. One pathological study in vivo showed that a sclerosant in a foam vehicle completely destroyed the intimal barrier after 2-min exposure, leading to endothelial edema, exfoliation from the tunica media, and thrombogenesis in the tunica media over 30 min [14]. Polidocanol is a relatively weak sclerosant compared with conventional liquid sclerosants, such as ethanol or EO [15], but foam formulations have the same immediate pathological effect on the endothelium as liquid sclerosants.

Polidocanol foam sclerotherapy has several potential advantages over conventional liquid sclerotherapy [16, 17]: (1) the sclerosant is injected as a gas and occupies the interior of the vessel without mixing with or being diluted by blood; (2) it is present as extremely small microbubbles, which increases the contact area with the vessel wall and subsequently the sclerotic effect in the desired area; (3) injection is generally painless and easily repeated. These factors also facilitate a significant reduction in the dose of sclerotic agent required, which subsequently reduces complications caused by sclerosant overdose.

Foam sclerotherapy has been indicated recently for vascular malformations [11, 12], and the number of visceral varices treated with transcatheter obliteration [13] has increased. However, symptomatic hepatic cysts are still generally treated by liquid sclerotherapy, although some of the advantages of foam sclerotherapy are applicable to hepatic cysts. Since the mid-2000s, several studies have shown that 5 % EOI is an efficient and safe alternative to ethanol for liquid sclerotherapy of symptomatic hepatic cysts. One single-center, nonrandomized study of three simple hepatic cysts and four simple renal cysts with an average volume of 328 (range 64–636) ml reported a mean reduction rate of 93 % (range 89–99 %) 3 months after a single session of 5 % EOI sclerotherapy, with no major complication [6]. Another study of 17 hepatic cysts (average volume, 589.8 ml; 26–1,885 ml) mainly associated with PLD reported a mean reduction rate of 88.4 % (47–100 %) following a single session of 5 % EOI sclerotherapy, for all but one cyst, and without any recurrence or major complications over a median follow-up period of 54 months (1 week–95 months) [7]. The mean reduction rate for the targeted cyst following polidocanol foam sclerotherapy was 97.9 % (97.7–98.3 %) in the present study, which is in no way inferior to that of previous reports.

Polidocanol foam sclerotherapy is more cost-effective than 5 % EOI sclerotherapy. There is no consensus on the maximum dose of 3 % polidocanol suitable for daily foam sclerotherapy sessions for hepatic cysts. Ten milliliters of 3 % polidocanol was tentatively determined as the maximum dose per session in the present study, because the manufacturer recommends that the maximum daily dose should not exceed 30 ml of 1 % polidocanol for endoscopic injection sclerotherapy to treat esophageal varices. The net dose of 3 % polidocanol per patient averaged 16 (range 8–30) ml in the present study. Thus, sufficient sclerotic effects were achieved with a lower dose of sclerotic agent than was the case for the two previous studies of EOI sclerotherapy, in which the treatment session dose of 5 % EOI was fixed to 10 % of the initial cyst volume and the mean amount of 5 % EOI required for sclerotherapy was 30.9 (range 6–60) ml [6] and 57.3 (range 10–120) ml [7], respectively.

In the phlebology field, reversible cardiac arrest [18] and transient neurological defects [19, 20] are rare but severe complications associated with foam sclerosants because of overdose and subsequent leakage to the systemic veins. No such complications were observed in the present study. Although a potential disadvantage of any sclerosant mixed with a higher proportion of gas is a reduction in visibility on fluoroscopy, preceding digital subtraction cystography with CO_2_ is useful for predicting the behavior of the injected gaseous sclerosant and can confirm that no sclerosant has entered the systemic circulation or adjacent hepatic vessels. Injected sclerosant is easily identified by C-arm CT after injection, as the air density area replaces fluid within the cyst. This also confirms the absence of migration into the surrounding hepatic vessels. One case of polidocanol foam injection-associated moderate fever was observed in this study, which is not much higher than that reported with EOI sclerotherapy [6, 7]. Delayed intracystic hemorrhage, a late complication in ethanol sclerotherapy [21], was not detected in this study.

Completion of sclerotherapy over a single session is ideal, considering that long-term implantation of a catheter increases the risk of infection and discomfort. The mean number of treatment sessions for sufficient sclerotherapy in this study was two sessions. Our final case (Case 3) was successfully treated with only a single session despite having a cyst with a relatively large initial volume (1,390 ml). This suggests that our first two cases may have been resolved with fewer sessions.

The study limitations were (1) its single-arm retrospective design with a small number of patients from a single institute without any randomization and (2) the relatively short follow-up period.

## Conclusions

C-arm CT-assisted polidocanol foam sclerotherapy following digital subtraction cystography with CO_2_ is a safe and effective treatment for symptomatic hepatic cysts that provides sufficient cyst volume reduction without severe complications. Further evaluation to determine the optimal dose and timing of polidocanol foam sclerotherapy relative to cyst volume are warranted, as are randomized controlled studies with long-term follow-up comparisons with EOI and sodium tetradecyl-sulfate foam.
